# Study protocol for a randomised pilot study of a computer-based, non-pharmacological cognitive intervention for motor slowing and motor fatigue in Parkinson’s disease

**DOI:** 10.1186/s40814-018-0375-4

**Published:** 2018-12-26

**Authors:** Joshua S. Payne, John V. Hindle, Aaron W. Pritchard, R. Rhys Davies, Rudi Coetzer, Giovanni D’Avossa, R. Martyn Bracewell, E. Charles Leek

**Affiliations:** 1 School of Psychology, Bangor University, Brigantia Building, Penrallt Road, Bangor, Gwynedd LL57 2AS UK; 2 Department of Care of the Elderly, Betsi Cadwaladr University Health Board, Llandudno Hospital, Conwy, UK; 3 Research and Development Office, Betsi Cadwaladr University Health Board, Bangor, UK; 4 The Walton Centre NHS Foundation Trust, Liverpool, UK; 5 North Wales Brain Injury Service, Betsi Cadwaladr University Health Board, Colwyn Bay, UK; 6 School of Psychology, Institute for Life and Human Sciences, University of Liverpool, Liverpool, UK

**Keywords:** Parkinson’s disease, Cognition, Computerised cognitive training, Non-pharmacological intervention, Motor symptoms, Fatigue, Feasibility trial

## Abstract

**Background:**

Parkinson’s disease (PD) is a chronic, neurodegenerative disorder affecting over 137,000 people in the UK and an estimated five million people worldwide. Treatment typically involves long-term dopaminergic therapy, which improves motor symptoms, but is associated with dose-limiting side effects. Developing effective complementary, non-pharmacological interventions is of considerable importance. This paper presents the protocol for a three-arm pilot study to test the implementation of computer-based cognitive training that aims to produce improvements or maintenance of motor slower and motor fatigue symptoms in people with PD. The primary objective is to assess recruitment success and usability of external data capture devices during the intervention. The secondary objectives are to obtain estimates of variance and effect size for changes in primary and secondary outcome measures to inform sample size calculations and study design for a larger scale trial.

**Methods:**

The study aims to recruit between 40 and 60 adults with early- to middle-stage PD (Hoehn and Yahr 1–3) from National Health Service (NHS) outpatients’ clinics and support groups across North Wales, UK. Participants will be randomised to receive training over five sessions in either a spatial grid navigation task, a sequential subtraction task or a spatial memory task. Patient-centred outcome measures will include motor examination scores from part 3 of the UPDRS-III and data from movement kinematic and finger tapping tasks.

**Discussion:**

The results of this study will provide information regarding the feasibility of conducting a larger randomised control trial of non-pharmacological cognitive interventions of motor symptoms in PD.

**Trial registration:**

ISRCTN, ISRCTN12565492. Registered 4 April 2018—retrospectively registered, in accordance with the WHO Trial Registration Data Set.

**Electronic supplementary material:**

The online version of this article (10.1186/s40814-018-0375-4) contains supplementary material, which is available to authorized users.

Parkinson’s disease (PD) is a chronic, neurodegenerative disorder affecting over 137,000 people in the UK and an estimated five million worldwide [1]. It largely reflects dopaminergic cell loss in the basal ganglia and consequent disruption to ganglia-thalamo-cortical motor circuits, including the supplementary motor area [2, 3]. The primary symptoms of PD are motor in nature (bradykinesia, tremor, rigidity, balance and gait disturbance), but are known to be accompanied with, and at times preceded by, cognitive impairments, including psychomotor slowing [4]. In the absence of a cure for the illness, a major challenge is to find interventions to ameliorate these debilitating motor symptoms. For most people with PD, treatment typically involves long-term dopaminergic therapy, which is associated with a range of dose-limiting complications, and considerable healthcare costs (which exceed £3.3b annually in the UK alone [5]). In this context, the prospect of developing effective complementary, non-pharmacological interventions is of considerable importance [6, 7]. Such interventions offer the prospect of better long-term clinical management of motor symptoms, improving quality of life and reduced healthcare costs.

The goal of this study is to begin to address this challenge by piloting a novel, hypothesis-driven, non-pharmacological intervention to improve motor function and diminish motor fatigue in PD. This novel approach is based on the use of hypothesis-driven cognitive task interventions to stimulate the motor system and ameliorate motor symptoms. It is important to note that this technique differs from cognitive stimulation (or so-called brain training) interventions that aim to increase resilience to cognitive—rather than motor—decline in illnesses such as dementia. Physiotherapy and occupational interventions including motor cueing used in conjunction with walking aids, gait training, regular exercise and dance can produce benefits for people with PD [8–10]. There is also a growing interest in the development of other approaches to the treatment of motor symptoms including, for example, neuro-feedback training based on real-time functional brain imaging [11–13], and other techniques based on cortical excitation induced by transcranial magnetic stimulation (TMS) [14–16]. These latter techniques, whilst showing considerable promise, are limited in a number of ways: They are unlikely to be cost-effective for large numbers of participants, and their suitability is limited to people who are able to undergo scanning (or procedures such as TMS), for whom these methods pose no health risk, and to individuals who are willing to undergo the procedure. To our knowledge, there have been no systematic attempts to investigate the potential use of targeted cognitive-behavioural interventions for the treatment of motor dysfunction. However, advances in our knowledge about the functional neuro-anatomy of the brain, and of the underlying neuropathology of PD, suggest that such an investigation is well motivated on scientific grounds, is timely and has the potential to deliver significant healthcare benefits.

This novel approach is inspired by recent neuroscience insights into the functional links between the supplementary motor area (SMA) (a key component of the motor system in the human brain) and specific cognitive processes. The background to the current project stems from an established body of work from our own, and other research groups, on the neural substrates of high-level visuospatial processing in the neurologically normal brain [17–20]. Previously, we have shown that visuospatial processing is supported by a network of regions including the posterior parietal cortex (PPL), dorso-lateral prefrontal cortex (DLPFC) and the SMA. This is most clearly shown by functional imaging studies of brain activity during the performance of visuospatial tasks like mental rotation or mental grid navigation [21].

Several studies, including work in our own lab, have shown that people with PD are impaired in visuospatial tasks like mental rotation and grid navigation [22, 23]. Furthermore, the magnitude of impairment in mental rotation is dependent on angular disparity: people with PD show slower rates of mental rotation consistent with impairment to visuospatial processes. This is consistent with what we know about the underlying neuropathology of PD. PD is linked to dopaminergic nigrostriatal cell loss and deregulation of neurotransmitter activity in the nigrostriatal pathways of the basal ganglia. This is thought to disrupt basal ganglia-thalamo-cortical motor circuits including the SMA [24].

A key theoretical question is why visuospatial tasks like mental rotation, and grid navigation, should activate regions such as the SMA—an area traditionally associated with the planning and execution of movement [17, 25]—even when the tasks are configured to require no overt motor response [21]. One hypothesis is that visuospatial tasks, and prehensile movement, draw on certain shared underlying functional processes that are supported by an overlapping cortical network including the SMA. For example, both tasks involve spatial transformation, that is, the computation of mappings between spatial locations. In the case of movement, this is required to calculate the direction and distance (i.e. vector) between the current hand location and a to-be-grasped object (e.g. when reaching for a cup). During mental rotation (or grid navigation), the same abstract computation may be used to calculate the angular distance between two features of a rotated object or the direction and distance required to map from one grid location to another. Interestingly, other evidence supporting this putative functional link between movement and visuospatial processing comes from behavioural studies showing interference between simultaneous performance of manual and mental rotation tasks [26, 27] and from studies of visuospatial processing impairments in PD [22, 23, 28]. By targeting these cognitive processes, we can, in principle, stimulate functioning of these regions and ameliorate motor performance.

We have previously examined this possibility in a local NHS-funded pilot study (BCUHB 05/WNo03/27; unpublished). Sixteen people with early-stage (H&Y 1/2) PD, tested during the ‘ON’ phase, with age-matched controls, completed a visuospatial ‘intervention’ task of mental grid navigation and a control task of sequence memory, in two separate sessions, a week apart. Immediately before and after the two tasks, we obtained precise measures of movement onset delay and velocity with a simple reaching task completed on a touch-sensitive screen. There were no significant changes in movement onset time, or velocity following either tasks, in the control group. However, the people with PD showed a reduction in motor performance in the simple reaching task (slower mean onset and velocity) following the control task, but an enhancement of motor performance following the visuospatial task (faster mean onset and velocity). This pattern was found in 12/16 (75%) participants. These data provide some evidence that the performance of cognitive tasks that involve visuospatial processing—even after a single 20-min session—can enhance resilience to motor slowing and improve motor function, reducing hypokinesia and bradykinesia. As a form of hypothesis-driven cognitive intervention for motor dysfunction, this approach could potentially have significant clinical benefits for people with PD. If effective, the approach could form the basis of a low-cost, non-pharmacological, treatment that could be made accessible to large numbers of people in the form of a portable software/web-based programme of exercises for home use.

Before the intervention can be considered for a large-scale clinical trial, we first need to better understand the size and robustness of effects in larger samples and assess its clinical significance—whether the intervention leads to levels of improvement in movement onset and velocity that are of benefit to people with PD and whether the benefits to motor function extend to other aspects of the illness such as reduced rigidity and increased motor fluency.

This pilot trial will examine these issues and further our understanding of the potential of this novel approach to the treatment of motor dysfunction in PD. This research will form a vital step in evaluating the clinical significance of the approach, its suitability as an intervention for people with PD and the feasibility of a future large-scale clinical trial.

## Aims

The primary aim of this pilot study is to test the implementation of a computer-based cognitive training intervention designed to impact on motor slowing and motor fatigue symptoms in people with PD. We aim to explore the impact of exposure to five sessions of a spatial grid navigation intervention task in the hope that it could improve these symptoms, relative to two other control tasks: sequential subtraction and spatial memory.

### Primary objective

Feasibility for a larger scale RCT will be determined based on the following:

- *Recruitment*: Successful recruitment and retention of a minimum of 30 participants for the full intervention within a 12-month funding period (currently reduced to 6 months active testing/recruitment, following IRAS application and successful research passport application). If this is not possible, we would need to re-evaluate recruitment practices and adjust the timeline of future studies to reflect the challenges faced. This may require identification of additional resource implications in terms of researcher/staff time, costings, length of the intervention and delivery methods.
- *Acceptability/usability*: Evaluation of the usability of the external devices used to capture movement kinematic (button box) and finger tapping data (modified touchscreen gloves). In evaluating utility of custom data capture devices, we will pay particular attention to any failures/limitations in data capture (e.g. proportional data loss, reliability/consistency of measures). Additionally, we will pay attention to any difficulties that participants may experience using the equipment following informal discussion at the close of the study that may aid in the re-design of key equipment. This will be necessarily open-ended and will not rely on formal qualitative analysis.

### Secondary objective

Estimation of variance and effect sizes to inform power calculations for larger scale RCTs for primary and secondary outcome measures:

- A clinically relevant change in UPDRS-III motor examination scores of at least 5 points from pre to post measurements in response to spatial grid navigation training relative to the other control tasks.
- Improvement in movement kinematic response times over the course of the five intervention sessions in response to spatial grid navigation training relative to the other control tasks.
- Improvement in motor fatigue measures over the course of the five intervention sessions in response to spatial grid navigation training relative to the other control tasks.

## Methods

### Study design

**Fig. 1:**
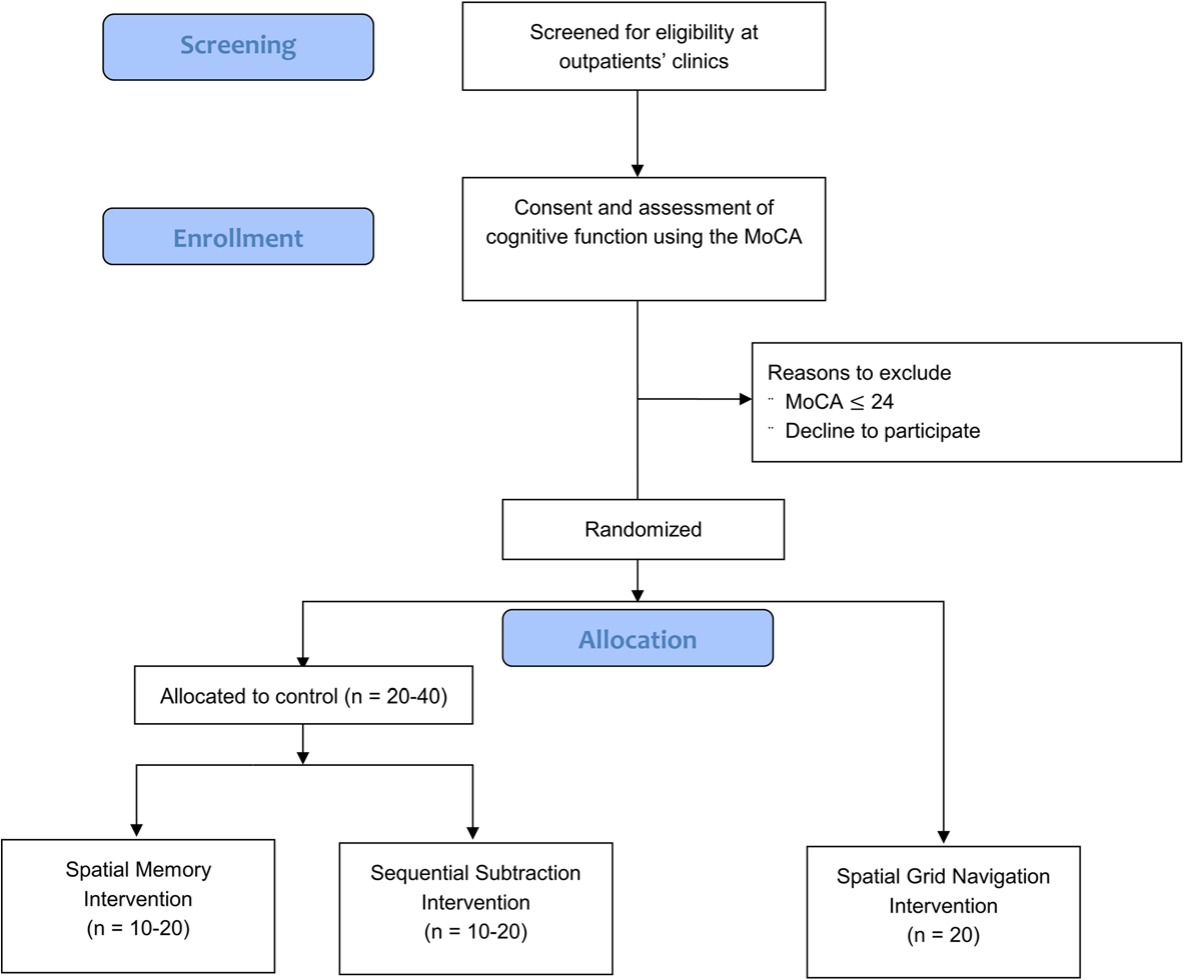
Protocol study flow diagram

We aim to conduct a three-arm, parallel, single-blind, randomised pilot trial, comparing the effect of five sessions of training on one of three cognitive intervention tasks. An adapted CONSORT (Consolidated Standards of Reporting Trials) study flow diagram is presented in Fig. 1.

### Study settings

Participants will be recruited from Movement Disorders outpatients’ clinics (Betsi Cadwaladr University Health Board, North Wales) and through direct contact following an earlier press release. We anticipate that most participants will prefer to be tested in their own homes, but all participants will be offered the option of being tested at local hospitals or at the School of Psychology, Bangor University. Participants will complete the study without modification to their medication or additional therapy regimens (e.g. physiotherapy, speech and language), and the study will be conducted to ensure maximum flexibility for all participants.

### Study participants

Adults with a diagnosis of Parkinson’s disease will be recruited for this study. The first participant enrolled in the study on 16/03/2018. To date, 10 (25% of 40) participants have completed the study and a further six have recently enrolled.

### Inclusion criteria

Participants of any age or sex are eligible to take part providing they meet the following criteria:

- Diagnosis of Parkinson’s disease in accordance with UK Brain Bank Diagnostic Criteria [29]
- Hoehn and Yahr stage 1–3 [30]
- Ability to give informed consent
- Montreal Cognitive Assessment [31, 32] score > 24

### Exclusion criteria

- Clinical diagnosis of dementia
- History of other significant neurological conditions
- Presence of visual hallucinations
- Cognitive impairment (MoCA score ≤24)
- Significant visual impairment affecting viewing of a computer screen

### Primary clinical outcome measures

The change in the motor examination score from the Unified Parkinson’s Disease Rating Scale - III (UPDRS - III; [33]) from Session 1 to Session 5.

### Secondary outcome measures

- *Motor fatigue*: Changes in the finger tapping count, the time between taps (kinesia measures) and tap dwell time (akinesia)
- *Motor slowing*: Changes in the response latencies (total movement time, movement initiation time and velocity) on the movement kinematic task

### Participant enrolment

Clinical determination of eligibility for the study will be assessed by Dr. John Hindle (JVH), a Consultant Geriatrician with expertise in movement disorders, and a team of other movement disorder specialists. Initial contact with eligible participants about the study will be made by a member of the research team (JP) present at clinics or via phone. In the absence of JP at clinics, the clinical studies officer and/or clinician will seek consent from interested participants to pass contact details onto the research team. Participants will have a minimum of 72 h to consider the study information once they have received the participant information sheet. An initial appointment will be arranged for participants to give informed consent, to complete the MoCA [31, 32] and other background questionnaires.

### Randomisation

Unique participant ID numbers were generated using IDGenerator software [34] with a 2:1:1 ratio for Grid Navigation to Subtraction to Spatial Memory (respectively *n* = 20, 10, 10) tasks for the initial 40 participants with the aim of achieving a 1:1:1 ratio dependent on recruitment success (*n* = 20 per treatment). ID numbers were randomised based on a sequence of random numbers. Participants were allocated an ID number based on this pre-defined allocation order at the point of giving informed consent. ID numbers and allocation to condition for participants 41–60 will be generated using the same protocol, ensuring that participants are blind to the content of other conditions until debriefing. JP is responsible for generation, allocation and assignment to interventions.

#### Blinding

This study is conducted single blind, in the sense that participants will not be made aware until debriefing which of the intervention conditions was expected to affect movement kinematic and motor fatigue symptoms. Double blinding is almost impossible in a cognitive intervention of this nature where the researcher is responsible for the setup and delivery of tasks in the presence of the participant. Data analysis will not be undertaken by an independent statistician, but once data has been compiled, we intend to pass anonymised raw data to an independent researcher who will generate new random IDs for all participants and revalue the intervention conditions. Our colleague will retain the linked information for all groups and new ID numbers until data analysis is completed.

### Intervention

Participants will be randomised to receive training over five sessions on Spatial Grid Navigation, Sequential Subtraction or Spatial Memory intervention tasks. Sequential Subtraction activates SMA to a similar extent as Spatial Grid Navigation, and we might expect that the sequential processing component could generalise to produce a similar improvement in movement kinematic and motor fatigue symptoms that we observed in an earlier pilot study. The spatial memory task is visually identical to the other two tasks but does not include a visuospatial sequencing component, acting as a neutral baseline task. The time between sessions will be dependent upon the availability of participants, and we will endeavour to work around participants’ other commitments and activities, as flexibly as possible to maximise retention. The variability in the time between sessions is a potential variable of interest, as the relative intensity may moderate the effectiveness of the intervention. We anticipate that participants will prefer to be tested in their own homes, but we are able to test people in the School of Psychology or in offices at local hospitals.

### Assessment measures

**Fig. 2:**
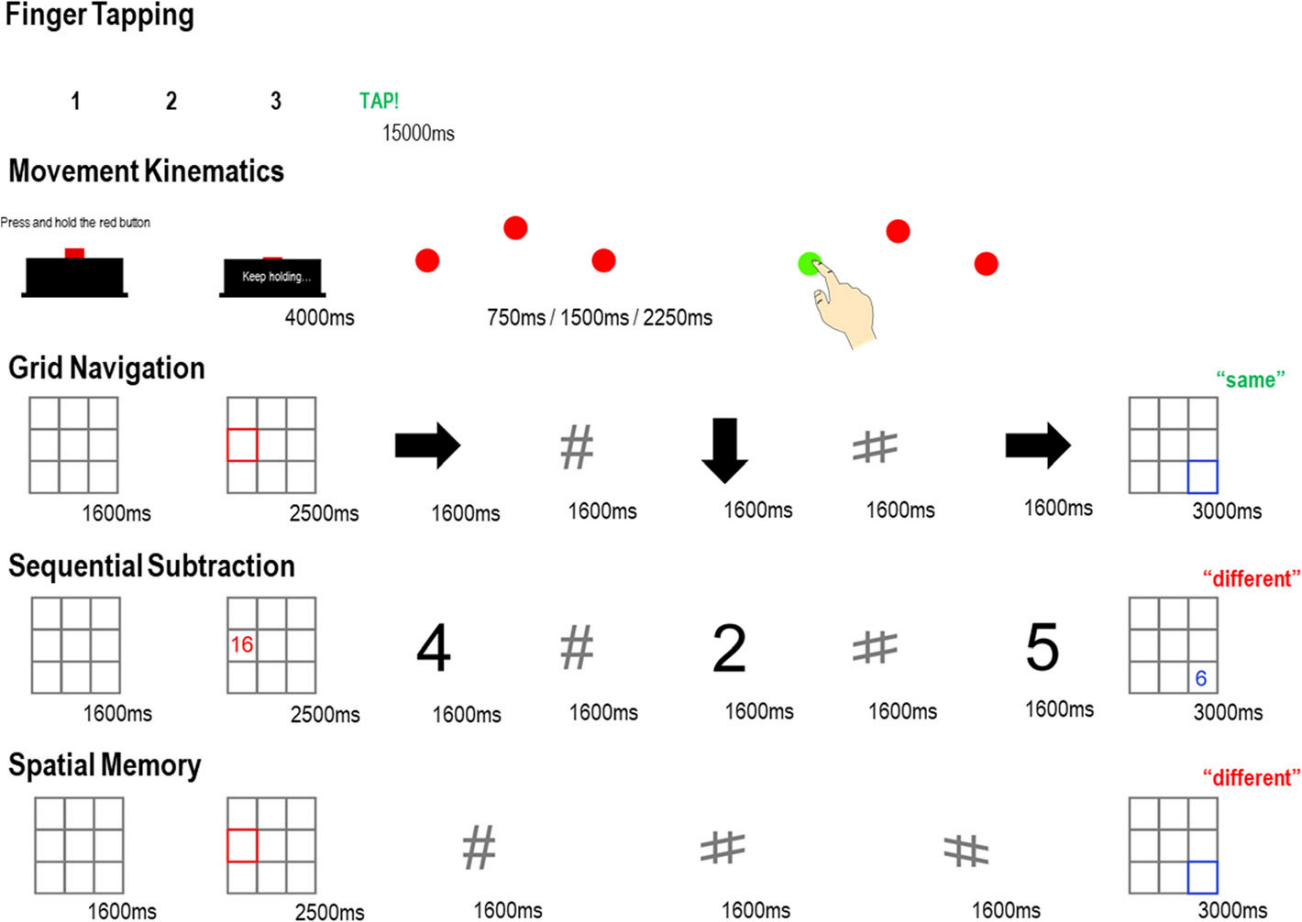
Schematic overview of each of the computer-based tasks

Figure 2 provides a schematic overview of computer-based tasks and intervention tasks.

#### Background measures

We will administer a series of background measures which enable us to provide a detailed description of our sample and individual symptomatic profile. In the first session, participants will give informed consent and will complete the following tasks and questionnaires:

- Montreal Cognitive Assessment 7.1 (MoCA; [31]). A score > 24 is needed to continue with the study. Participants will be told that we are looking for participants who score within a certain range to make sure that the tasks are suitable. If this is not achieved, participants will be debriefed and asked if they would like to be contacted about other ongoing studies and consent will be sought to relay their interest to the clinical studies officer. JP was assessed conducting the MoCA by a clinical neuropsychologist (RC), and all of the MoCA tests will be second-scored for inter-rater reliability by RC.
- Demographics questionnaire: sex, education, employment history and experiences of PD symptoms.
- Edinburgh Handedness Inventory [35] as a measure of hand dominance, to account for known advantages in motor tasks for the dominant hand and as an additional potential moderator of performance if the dominant hand is most affected by PD.
- Parkinson’s disease quality of life—short form (PDQ-8 [36]) as a general measure of quality of life.
- Parkinson’s Fatigue Scale (PFS-16 [37]) as a global measure of fatigue.
- Non-motor symptoms questionnaire (NMS [38]) to provide a global overview of PD symptomatology outside of the motor domain. The severity of non-motor symptoms could influence the efficacy of the intervention.
- Parkinson’s Disease Sleep Scale (PDSS [39]) to measure overall perceived sleep quality, which contributes to cognitive performance.

### Primary objective: feasibility assessment

#### Recruitment

Recruitment refers to the total number of participants who completed five intervention sessions. To determine feasibility, we expect a minimum of 30 participants to have been recruited and completed the sessions by the close of the study period.

#### Acceptability/usability

Usability will be assessed in terms of the fidelity of the data. We will report proportional data loss for each of the secondary measures outlined below and as consistency/reliability of the captured data. A high proportional data loss or low reliability would indicate a need for revision of device designs or protocols in explaining their use. In terms of acceptability, we will pay particular attention to any difficulties that participants may face over the course of the study and conduct an informal discussion to gather opinions about potential improvements. However, because of the lack of expected depth in these responses, we do not intend to conduct formal qualitative analyses.

### Secondary objectives

#### Primary clinical outcome measure

The motor examination from part 3 of the Unified Parkinson’s Disease Rating Scale-III [33] will be the primary clinical outcome measure for this study. The UPDRS will be administered to all participants by JP at the beginning of experimental session 1 and the end of experimental session 5. JP has received training and supervision at outpatients’ clinics from JVH. UPDRS motor assessment at both time points will be video recorded for all participants and 20% will be blind-rated by a neurologist (GD) for inter-rater reliability, excluding scores for tone/rigidity.

#### Secondary patient-centered outcome measures

Both secondary measures will be assessed before and after the intervention task administered in each session. All tasks are coded in OpenSesame 3.2.1 [40] and run on a Dell Inspiron 15 5000 series touchscreen laptop (1920 × 1080 resolution, 15.6″ screen). Hand/arm order was counterbalanced between administrations, within sessions (e.g. pre: L-R; post: R-L) and between tasks (e.g. finger tapping L-R; pointing R-L).

##### Finger tapping

Finger tapping performance is used routinely in clinical practice to assess the presence and severity of bradykinesia in people with PD but is often restricted to the count/number of taps, which provides only limited information (e.g. [41]). Objective, multivariate measures of tapping performance that quantify the speed and variability of performance as well as tap frequency are needed in addition to clinical assessment. Our measures of finger tapping are influenced by those collected from the recently developed BRAIN (BRadykinesia-Akinesia INcoordination) test, recently validated for assessment of bradykinesia in PD [42, 43]. At the beginning of the trial, the BRAIN test was not available for general use. As such, we collect detailed response data from the standard single-digit finger tapping task included as part of the UPDRS-III, using pairs of modified touchscreen gloves. These gloves are a practical, low-cost measurement device that preserves natural motion. Each contact of the conductive pads on the thumb and index finger registers as a left or right mouse click for the respective hand. Each trial begins with a 3-s countdown, followed by the word ‘TAP!’ which cues the action and stays on the screen for 15 s. Each hand will be tested separately with the wrist resting against the table and the other hand laid flat. Kinesia is measured as the number of taps over 15 s and the time between taps. The dwell time for each tap is collected, as a measure of akinesia. Lower values or increased variance for kinesia measures would reflect worse performance, indicating more motor fatigue, whereas lower values for akinesia measures would reflect less motor fatigue, as participants are able to reliably disengage from the movement. We will also be able to calculate a slope of performance for each hand as a profile measure for the number of taps per second as a more specific measure of fatiguing bradykinesia.

##### Movement kinematics

In people with PD, movement kinematic measures of velocity and trajectory are impaired relative to healthy controls [44, 45] and changes in the measures in response to an intervention may index subtle targets for rehabilitative efforts. In our simple pointing task, participants must press down and hold a button on a custom-made USB response box for 4000 ms. Three red circles, 2° (102px, 57 cm) in diameter, are displayed in an arc at left [(− 750px, − 47px) ≈ (13°, 0.85°) from centre], centre [(0px, − 325px) ≈ (0°, 6°)] and right locations [(750px, − 47px) ≈ (13°, 0.85°) from centre]. Participants continue to hold down the response button until one of the circles turns green at a variable SOA of 750 ms, 1500 ms and 2250 ms. Participants will be instructed to make a reaching movement to touch the green target as fast and as accurately as possible, before returning their hand to the response box (they do not have to touch the button to end the trial). Each participant will wear a pair of touchscreen-compatible gloves to neutralise differences in skin conductivity. To minimise strain on the wrist, a mousepad with gel wrist support will be placed under the response box. There are 18 trials per hand. The primary outcome measure on this task is action completion time (touch response time − target onset time) and was the primary dependent variable in our pilot study using an analogue version of the task. The action completion time can be decomposed into two separate elements: movement initiation time (key release time − target onset time) and reaching time/velocity (touch response time − initiation time), which could reflect subtle targets for improvement of motor slowing in adults with PD. In addition, we collect measurements of accuracy of the touch response and deviation from the centre of the target on each trial as a measure of incoordination.

### Intervention tasks

Target positions in the start and test grids of the tasks described below were identical across tasks. Five independent blocks of trials were generated and counterbalanced between sessions and participants using a 5 × 5 Latin square to mitigate any potential bias in the sequence generation process. Two blocks of 18 trials are administered with a self-paced break in between. All tasks were programmed in OpenSesame 3.2.1 [40]. To avoid contamination of motor fatigue and motor slowing measures, responses are made verbally in all three intervention tasks. Responses are recorded by OpenSesame via a small cardioid lapel microphone and stored as individual wave files. Response times are then extracted offline using the VoiceKey programme for Windows command line [46] or manually verified using Praat [47] in the case of any errors/noise spikes.

#### Spatial grid navigation

On each trial, participants see an empty grid made up of nine squares, subtending 10° visual angle for 1600 ms. A start square, highlighted in red, is displayed for 2500 ms, and participants are told to hold the position of the start square in memory. A sequence of five screens are displayed (1500 ms + 100 ms blank ISI) showing sequences of two, three or four arrows (subtending 4°), interspersed with grey hash marks as placeholders. The arrows indicate movement of the red starting square by one space in any of the four cardinal directions (up, down, left, right), restricted within the boundaries of the grid. No movements are made to the placeholders. Participants must track the position of the red square based on the observed sequence, in their minds eye. At the end of the sequence, a test grid is presented with a target square highlighted in blue. Participants must decide if the position of the blue test square is consistent with the final position of the presented sequence (50:50 same-different). Participants make a vocal ‘same’ or ‘different’ response whilst the test grid is on the screen (3000 ms).

#### Control group A: sequential subtraction

A minimum of 10 (up to maximum of 20) participants will be enrolled into this arm of the study, dependent on recruitment success. The trial sequence for this task is identical to the spatial grid navigation task with a few modifications. The start square is replaced with a number highlighted in red. The arrows in the sequence were replaced with the numbers 1–9, which had to be sequentially subtracted from the start number before deciding whether the test number, presented in blue, was the same or different as the final total in the sequence. Sequences varied in length from two to four numbers, plus between one and three placeholders.

#### Control group B: spatial memory

A minimum of 10 (up to maximum of 20) participants will be enrolled into this arm of the study, dependent on recruitment success. This task contains no spatial sequencing component and will provide a pure baseline for the present study. In this task, participants see a start square, highlighted in red, and are told to hold its position in memory for a duration of two to four successive placeholders (1600 ms ea.) before a test grid appears with a square highlighted in blue. Participants are instructed to verbally report whether the test grid is in the ‘same’ or ‘different’ position to the start grid.

### Procedure

This protocol was written in accordance with the CONSORT [48] SPIRIT guidelines (Standard Protocol Items: Standards of Reporting Trials) [49] for pilot and feasibility trials and the TIDieR guidelines (Template for Intervention Description and Replication) [50] for reporting of complex interventions. A SPIRIT table is presented as Table 1, and the SPIRIT and TIDieR checklists are available as Additional files 1 and 2. When the results are known, we will submit the final manuscript in accordance with the CONSORT and TIDieR guidelines. JP is a research project support officer with a background in experimental psychology and clinical interventions in stroke. JP will be responsible for the organisation and delivery of the intervention, including scheduling participants, gaining written informed consent, face-to-face task administration, data management and debriefing. Following initial contact, participants will be given a minimum of 72 h to consider whether they would like to take part. Participants give informed consent and complete the MoCA and background assessments in the initial session (− *t*_1,_ SPIRIT table), which will take no longer than 1 h. Each participant will take part in five intervention sessions with a minimum frequency of approximately one session per week. The time between sessions will vary and is dependent on the availability of individual participants. We take a flexible approach to encourage engagement and reduce attrition. Providing there is sufficient variability, we intend to explore the effect of intervention intensity as a moderator in our analyses. The motor examination from part 3 of the UPDRS-III will be administered at the beginning of session 1 (*t*_1_) and the end of session 5 (*t*_5_). Secondary movement kinematic and finger tapping measures will be administered before and after the administration of the intervention tasks in every session and presented in a fixed order of finger tapping, followed by movement kinematics. In each session, we will collect information on time since last dose of medication, hours and quality of sleep (1 = very poor; 10 = excellent). A rating of fatigue (1 = no fatigue/full of energy; 10 = absolutely drained) will be taken at the beginning of each task to monitor within and between-session fluctuation. Session 1 and session 5 will take up to 75 min to complete, dependent on breaks and progress through tasks, but sessions 2–4, which include only computer tasks, will take between 45 and 60 min per session. Participants will be debriefed and offered the opportunity to provide feedback on the intervention/tasks at the end of session 5.

**Table 1:**
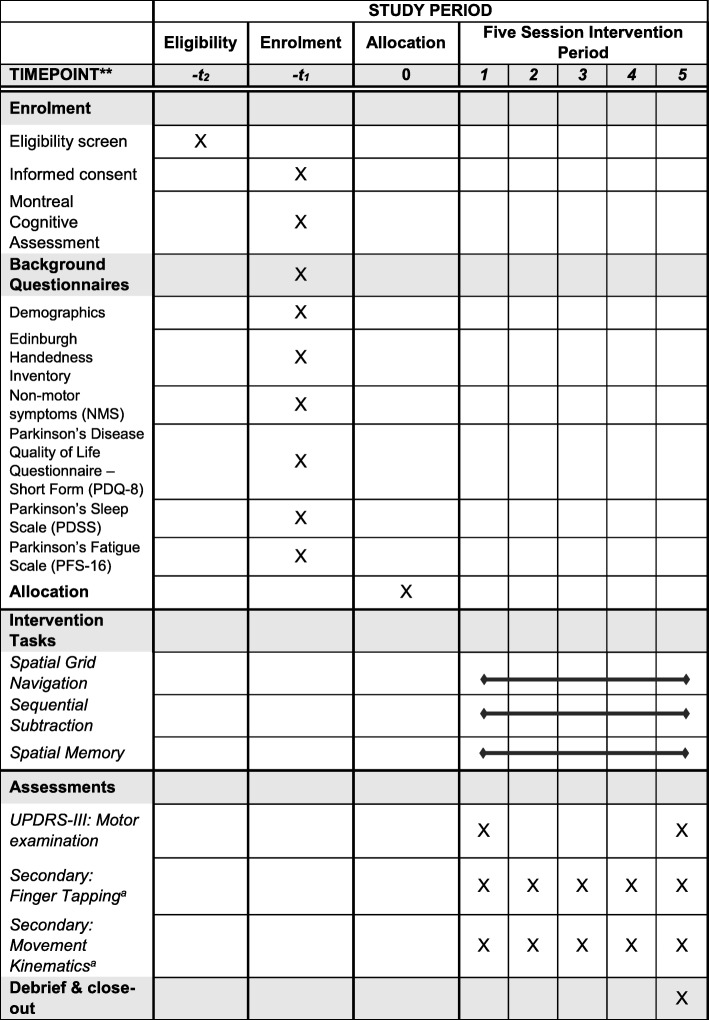
Schedule of enrolment, interventions and assessments

^a^Delivered twice per session, pre and post intervention tasks. Finger tapping is always administered before movement kinematics

**The time between sessions will be variable, in keeping with participants’ schedules and other commitments

### Sample size

The target sample size for this study is between 40 and 60 participants. On the basis of our previous work, we estimate an attrition rate of 20%. Participants who are unable to complete the study or withdraw for any reason will be replaced. The target sample estimate is based on a maximum sample that we can reasonably expect to obtain over a 9–12-month active recruitment period, given the population and both clinical and research resources available.

### Statistical methods

Summary statistics for demographic and background information will be reported for all participants in each of the three groups. Presented figures will endeavour to provide an overview of individual data points as well as measures of central tendency, variance and 95% confidence limits. Exploratory analyses exploring the relationships between participant characteristics, intervention intensity and primary/secondary outcomes will be conducted to inform future work. With sufficient participants, mixed effects analyses will be applied to secondary outcome measures and intervention data, to identify potential patterns of effects, which will enable us to characterise both within- and between-subject variances. The analyses are primarily designed to support descriptive analysis of data and inform future power calculations for analyses of this type. Interpretation of patterns of data will be cautionary and based upon estimates of confidence to guide future hypothesis testing.

### Data handling and storage

Identifying participant information will be stored in a password-protected database on an external, encrypted hard disc. Additionally, the IDgenerator [34] software creates layered ID numbers linking an ID paired with identifying information in a separate password-protected database to an ID number associated with study data using a third, temporary ID. At the close of the study, this link will be erased and identifying information will be destroyed. Over the course of the study, electronic data will be stored on a password-protected laptop with an active firewall and anti-virus. Regular backups of electronic data will be made on an encrypted external hard drive and automatically uploaded to a protected web server. During transport between sites, anonymised participant packs and the laptop will be locked in a file box, in a locked car boot. Consent forms and completed participant packs will be stored in a locked file cabinet in a locked office at Bangor University. All data will be kept for 10 years following final access.

### Auditing

The project will be subject to independent auditing by Betsi Cadwaladr University Health Board as the funding body and by Bangor University as the sponsor. The trial team will meet quarterly to discuss the trial progress and/or modifications. Regular updates will be sent out to all members of the team, as well as the clinical studies officer assisting with recruitment.

### Harms

This is non-Clinical Trial of an Investigational Medicinal Product (non-CTIMP) and as such poses minimal risk to participants, and it is not necessary to institute a formal data management committee. We do not anticipate any adverse or serious adverse events will arise during the course of the study. Reporting of such events will be carried out in accordance with NHS policies. Worsening symptoms of PD, hospital stays for elective treatment for pre-existing medical conditions or death resulting from PD will not be reported as SAEs.

### Protocol version

This paper is based on protocol version 4.0 (30/01/2018). Any amendments will be communicated with the study team and addressed in a final published manuscript once results are known.

### Dissemination

The data will be presented at national and international conferences and published in peer-reviewed journals. The headline results of the study will be shared with participants at the close of the study and made available to the wider public through national bodies (e.g. Parkinson’s Disease UK).

## Discussion

The protocol described is hypothesis-driven, founded on previous pilot data from an independent group of people with PD and underpinned by a considerable body of work which supports the link between activation of the SMA and visuospatial processing. Our previous data demonstrated a significant improvement in measures of movement kinematics and motor fatigue following a single administration of the spatial navigation task. However, the question remains whether repeated exposure to the same intervention would result in a cumulative improvement or support maintenance of current functioning, providing the basis for the future development of this non-pharmacological cognitive intervention. If we observe the expected effects, we would utilise the data for a formal power calculation to inform a large-scale RCT. The longer-term view is to develop an evidence-based, online or app-based platform that could be accessed by people in their own homes alongside other pharmacological or physical treatments to support maintenance of motor slowing and motor fatigue symptoms. An app-based or online portal would enable automatic allocation to intervention condition and complete double blinding of the intervention conditions and concealment during data analysis stage.

As it currently stands, the study protocol is restricted to early-middle disease stage for adults with good cognitive function. This feasibility trial will enable us to refine procedures and task instructions to maximise accessibility and engagement with the tasks, which can be further developed for application to the broadest possible sample. In the face of favourable results, future directions should include longer term follow-up and tracking of motor decline in people with PD. The results from this study will provide valuable insight into the feasibility of cognitive interventions of this type and generate/refine hypotheses for future studies.

## Additional files

Additional file 1: SPIRIT 2013 Checklist. (DOC 121 kb)

Additional file 2: The TIDieR (Template for Intervention Description and Replication) checklist. (DOCX 31 kb)
